# Multimodal Identification of Molecular Factors Linked to Severe Diabetic Foot Ulcers Using Artificial Intelligence

**DOI:** 10.3390/ijms251910686

**Published:** 2024-10-04

**Authors:** Anita Omo-Okhuasuyi, Yu-Fang Jin, Mahmoud ElHefnawi, Yidong Chen, Mario Flores

**Affiliations:** 1 Department of Electrical and Computer Engineering, Klesse College of Engineering and Integrated Design, University of Texas at San Antonio, San Antonio, TX 78249, USA; anita.igberaese@utsa.edu (A.O.-O.); yufang.jin@utsa.edu (Y.-F.J.); 2 The National Research Centre, Cairo 12622, Egypt; mahef@aucegypt.edu; 3 Greehey Children’s Cancer Research Institute, University of Texas Health Science Center at San Antonio, San Antonio, TX 78229, USA; cheny8@uthscsa.edu

**Keywords:** diabetic foot ulcer, electronic health records, machine learning, risk factors, OCHIN database, healthcare analytics

## Abstract

Diabetic foot ulcers (DFUs) are a severe complication of diabetes mellitus (DM), which often lead to hospitalization and non-traumatic amputations in the United States. Diabetes prevalence estimates in South Texas exceed the national estimate and the number of diagnosed cases is higher among Hispanic adults compared to their non-Hispanic white counterparts. San Antonio, a predominantly Hispanic city, reports significantly higher annual rates of diabetic amputations compared to Texas. The late identification of severe foot ulcers minimizes the likelihood of reducing amputation risk. The aim of this study was to identify molecular factors related to the severity of DFUs by leveraging a multimodal approach. We first utilized electronic health records (EHRs) from two large demographic groups, encompassing thousands of patients, to identify blood tests such as cholesterol, blood sugar, and specific protein tests that are significantly associated with severe DFUs. Next, we translated the protein components from these blood tests into their ribonucleic acid (RNA) counterparts and analyzed them using public bulk and single-cell RNA sequencing datasets. Using these data, we applied a machine learning pipeline to uncover cell-type-specific and molecular factors associated with varying degrees of DFU severity. Our results showed that several blood test results, such as the Albumin/Creatinine Ratio (ACR) and cholesterol and coagulation tissue factor levels, correlated with DFU severity across key demographic groups. These tests exhibited varying degrees of significance based on demographic differences. Using bulk RNA-Sequenced (RNA-Seq) data, we found that apolipoprotein E (*APOE*) protein, a component of lipoproteins that are responsible for cholesterol transport and metabolism, is linked to DFU severity. Furthermore, the single-cell RNA-Seq (scRNA-seq) analysis revealed a cluster of cells identified as keratinocytes that showed overexpression of *APOE* in severe DFU cases. Overall, this study demonstrates how integrating extensive EHRs data with single-cell transcriptomics can refine the search for molecular markers and identify cell-type-specific and molecular factors associated with DFU severity while considering key demographic differences.

## 1. Introduction

Diabetes prevalence estimates in South Texas exceed the national estimate and the number of diagnosed cases is higher among Hispanic adults compared to their non-Hispanic white counterparts. Bexar County and the city of San Antonio report significantly higher annual rates of diabetic amputations than Texas despite reporting a similar prevalence of diabetes. In 2017, Hispanic adults in Bexar County were hospitalized for diabetic amputations at significantly higher rates (10.7/10,000) than non-Hispanic black (7.4/10,000) and non-Hispanic white (6.0/10,000) adults [1]. Diabetic foot ulcers (DFUs) are a severe complication of diabetes mellitus (DM), which is characterized by high blood glucose levels due to insufficient insulin. DFUs, which manifest as ulcers on the feet, lead to more hospitalizations than other diabetic complications and are the leading cause of non-traumatic amputations in the U.S. In 2023, about 5% of diabetic patients developed DFUs, and around 1% resulted in amputations [2]. In addition to this, studies have shown that other risk factors, such as previous amputation history and other risk factors, can increase the likelihood of amputation in DFU prognosis [3,4]

The Meggitt–Wagner system grades DFUs from 0 to 5 based on severity. Grade 0 indicates an intact foot at risk for ulcers, Grade 1 is a superficial ulcer, Grade 2 involves deeper structures, Grade 3 includes abscesses, Grade 4 involves gangrene in the forefoot, and Grade 5 includes gangrene of the entire foot [5]. Treatments range from wound care to amputation.

Risk factors for DFUs include diabetic neuropathy [6], peripheral vascular disease [7], previous ulcers, poor glycemic control, long-term diabetes, race/ethnicity, smoking, insulin use, poor vision, age, and sex [8]. The prevalence and severity of DFUs can vary significantly across demographic groups due to genetic, lifestyle, and socio-economic factors. Understanding these demographic differences is crucial for developing targeted interventions and improving clinical outcomes. Utilizing electronic health records (EHRs) and machine learning can improve DFU prediction and knowledge [9,10]. Electronic health records (EHRs) contain patient information in all forms and formats. Unstructured EHRs typically contain clinician notes, discharge summaries, and imaging interpretations and lack a predefined format. Structured EHRs, however, store data in predefined formats like tables, making information storage and retrieval systematic. These data include details such as birth and death dates, race, socioeconomic status, sex, and housing situation, providing a comprehensive view of the patient’s health.

Structured EHRs also use standardized coding systems to encode medical information. Common codes include the International Classification of Diseases, 10th Edition (ICD-10), for diseases and conditions; Current Procedural Terminology (CPT) for procedures; Healthcare Common Procedure Coding System (HCPCS); Systematized Nomenclature of Medicine—Clinical Terms (SNOMED-CT); and the National Drug Code (NDC). This project uses Logical Observation Identifiers Names and Codes (LOINC) to track and identify laboratory tests conducted during disease management [11]. Using EHR data and key demographic information, we aimed to understand the factors influencing diabetic foot ulcers (DFUs). 

**Figure 1:**
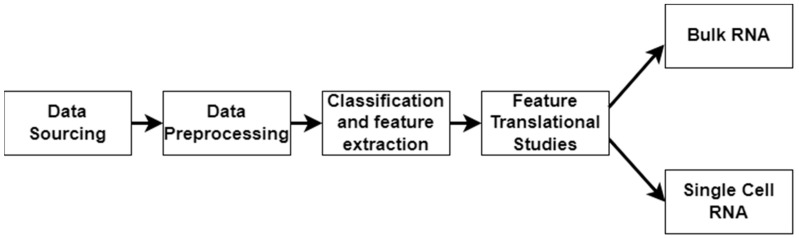
Workflow illustrating the stages of the multimodal approach. The pipeline consists of data sourcing, preprocessing, and classification/feature extraction, and feature translational studies, culminating in bulk RNA sequencing and single-cell RNA sequencing analyses.

In this work, we applied a multimodal approach to identify factors related to the severity of DFUs (Figure 1). We used LOINC codes from the EHR laboratory tests carried out on DFU patients. We continued our multimodal approach by analyzing bulk RNA and single-cell RNA sequencing datasets to identify molecular factors related to DFU severity and validate the findings derived from using the EHRs. Bulk RNA sequencing gives a broad overview of gene expression across many cells, while single-cell RNA sequencing reveals individual cell details and functions within tissues.

Several efforts have been made to use EHRs to identify factors related to disease severity. Adelaide M. Arruda-Olson et al. [12] developed a prognostic tool for patients with peripheral arterial disease utilizing automated data extracted from EHRs. This allowed for real-time and personalized risk prediction during patient care. Wang et al. applied this towards the early detection of diabetic retinopathy [13]. This developed predictive technology served as an early warning system, encouraging patients to undergo regular eye examinations for early screening and potential treatment of diabetic retinopathy. Hamid Safi et al. [14] also developed a method for the early detection of diabetic retinopathy. However, they explored the changes in protein expression as a diagnostic biomarker. The use of molecular changes in proteins and changes in the expression of the associated genes served as a bridge in our study between the findings in EHRs to our use of transcriptomics datasets.

In the fast-changing realm of diabetes research, transcriptomics has become vital for understanding the intricate molecular mechanisms behind the disease and its complications, such as diabetic foot DFUs, nephropathy, and retinopathy. The two main techniques in diabetes research are bulk RNA sequencing and single-cell RNA sequencing, which offer distinct insights. Bulk RNA sequencing gives a broad overview of gene expression across tissues, aiding in finding molecular factors and treatment targets in diabetic tissues. However, it lacks the ability to consider the different cell types present. On the other hand, scRNA-seq allows for a detailed examination of cellular compositions, pinpointing the roles of specific cells in disease processes. There have also been efforts to use the collaborative knowledge of bulk and single-cell RNA sequencing datasets and analyses in the identification of tumor immune microenvironment-related signatures [15], and in the construction of a stemness-related signatures for predicting prognoses and immunotherapy responses in hepatocellular carcinoma [16]. However, to the best of our knowledge, no studies have successfully incorporated clinical data from EHRs and transcriptomics in the identification of factors for severe DFUs.

Multimodal approaches significantly contribute to both the healthcare [17] and computational fields by filling important gaps in DFU severity prediction and management. In healthcare, we aim to identify factors of DFU severity that can be used for further investigation of biomarkers and to understand the molecular mechanisms involved in severe DFUs. Our approach links EHRs with transcriptomic data to reduce the search space in the low number of single-cell datasets due to their present cost. On the computational side, we introduce an innovative technique for combining structured clinical data from EHRs with bulk RNA sequencing and single-cell RNA sequencing datasets. By employing machine learning algorithms, we can effectively pinpoint key significant factors of DFU severity and progression. Using transcriptomics datasets, we further confirmed factors such as the *APOE* gene, which could guide customized interventions. Ultimately, this study established a model for incorporating clinical and molecular data for the identification of factors in chronic diseases.

The subsequent sections of this paper will present a detailed account of the findings derived from the dataset analysis (Section 2), followed by an in-depth discussion (Section 3) that contextualizes the results and their implications. Additionally, the conclusions of this study will be addressed in this same section. The methodology employed in this study will be expounded upon in Section 4.

## 2. Results

In this section, we divided the results to reflect the stages of the integration of EHRs and transcriptomics. Section 2.1 details the results from the EHRs, Section 2.2 details the results from bulk RNA sequencing, and Section 2.3 details the results using single-cell RNA sequencing data.

### 2.1. Analysis Using Electronic Health Records

We divided our results using the EHRs into two parts, as listed below.

#### 2.1.1. Data Preprocessing and Interpretation from EHRs

In this study, we processed electronic health record data and shed light on notable demographic distinctions. Our analysis started with a thorough analysis of EHR data consisting of 8969 de-identified patient records. To work with only the most relevant dataset, we meticulously filtered the dataset by categorizing patients into Type 1 diabetes, Type 2 diabetes, and an ‘other’ group, subsequently excluding entries under ‘other’ from our analysis.

**Figure 2:**
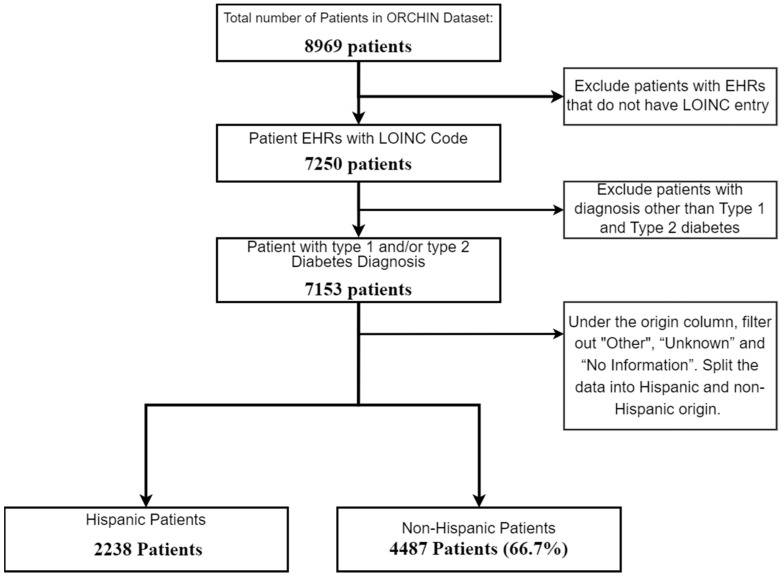
Flowchart of patient selection for study on diabetic patients by Hispanic origin.

Our focus was centered on understanding how demographic factors, such as age, sex, and socio-economic status, influence the molecular differences observed in the laboratory test results. This approach aimed to identify specific demographic variations that may contribute to the severity of DFUs, allowing for more personalized risk assessments and treatment strategies. These differences, influenced by genetic, lifestyle, and socio-economic factors, may have contributed to the varying likelihood of developing diabetic foot ulcer disease. To enhance the data quality, entries lacking laboratory data were excluded, leaving us with 7153 patient records for analysis. As our strategy aimed to identify factors related to severe DFUs in two demographic groups, we split the samples in the dataset into Hispanic and non-Hispanic subsets. Figure 2 below illustrates the initial filtering steps of the dataset.

The dataset also contained demographic information, which was used as labels for further feature selection, including vital status (alive or deceased), biological sex, income relative to the current Federal Poverty Line (FPL) (which gives insight into the economic power of the patients), whether the patient resides in a rural community, and whether they live in the northern or southern states of the United States. Analyzing these demographic variables alongside clinical data provided insights into their effects on disease development. In this work, vital status was particularly useful in defining severity.

In addition to demographic data, clinical laboratory test results are critical in identifying factors. EHRs contain LOINC codes that are used to track the laboratory tests conducted to diagnose and manage diseases. Our dataset included 63 such codes, which we used for further analysis. Given our focus on identifying differences in DFU diagnoses between Hispanic and non-Hispanic populations, we employed machine learning techniques, specifically Random Forest, to analyze the high-dimensional dataset and identify the most important factors.

Machine learning is essential for handling the large number of variables in this dataset, as manual methods would be inadequate for discovering complex relationships between demographic, clinical, and molecular data. Random Forest was chosen for its ability to handle both structured and unstructured data, making it ideal for the diverse formats found in EHRs. Additionally, Random Forest mitigates the risk of overfitting by aggregating decisions from multiple trees, providing more reliable predictions. This model is also more interpretable compared to other machine learning techniques, which is crucial for identifying key predictors of DFU risk.

**Figure 3:**
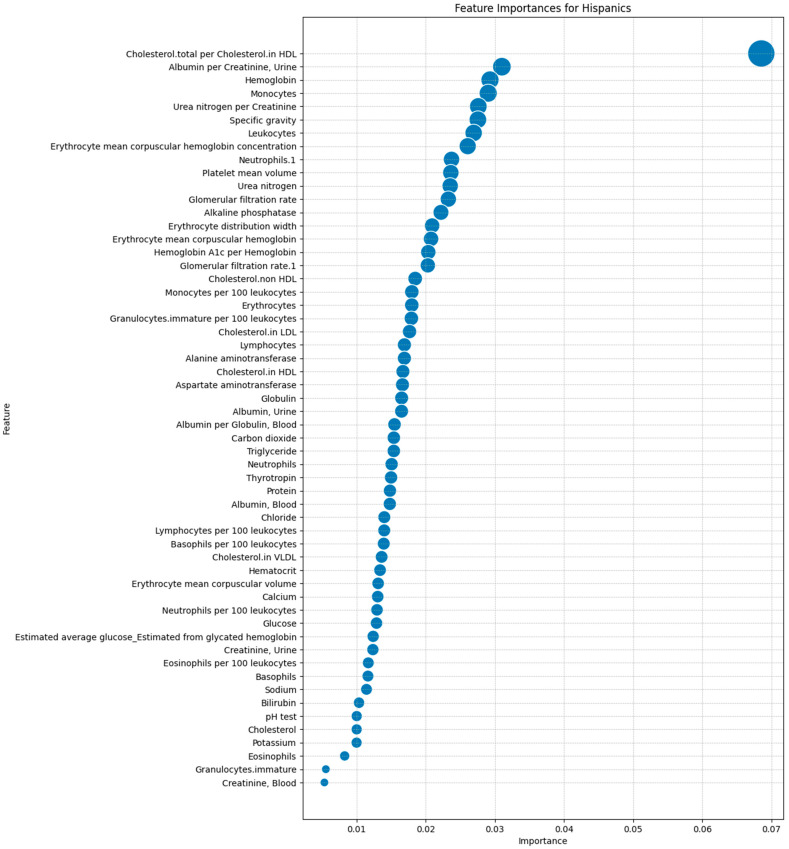
Feature importance rankings for laboratory tests in predicting the DFU outcomes in the Hispanic population using a Random Forest model.

After identifying the 63 laboratory tests in the dataset, we ranked them by importance using the Mean Decrease in Accuracy (MDA) metric from the Random Forest model. This allowed us to prioritize features based on their impact on predictive accuracy, ensuring that we focused on the most relevant tests that distinguished between Hispanic and non-Hispanic groups. Figure 3 illustrates the MDA rankings, with the most important feature being the total cholesterol to high-density lipoprotein (HDL) cholesterol ratio. Other significant features included the Albumin/Creatinine Ratio (ACR), hemoglobin level, and monocyte count, pinpointing the importance of renal function, oxygen transport capacity, and immune response in predicting DFU outcomes.

Once all the tests were ranked, we further looked for statistical differences between the Hispanic and non-Hispanic groups using the Mann–Whitney U test. 

By combining machine learning-driven feature selection with rigorous statistical testing, we identified specific molecular and demographic factors that are especially influential in DFU development. The laboratory tests that showed the most significant differences served as the focus for further analysis. Table 1 presents the tests with the greatest statistical differences. This integrated approach provides a comprehensive overview of the factors contributing to DFU severity and lays the groundwork for personalized interventions.

#### 2.1.2. Albumin/Creatinine Ratio Test as Basis for Assessing DFU Risk in Hispanics

**Figure 4:**
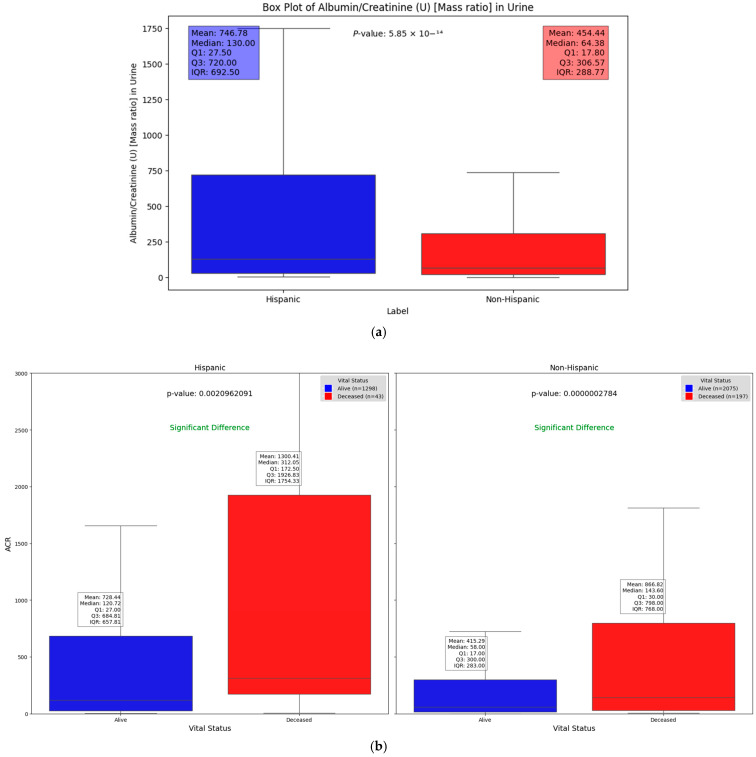
Box plots illustrating significant differences in Albumin/Creatinine Ratio: (**a**) demonstrates statistical difference between Hispanic and non-Hispanic groups as indicated by *p*-value of; (**b**) ACR differences between surviving and deceased individuals within the Hispanic and non-Hispanic population.

We observed significant differences in the Albumin/Creatinine Ratio (ACR) test results across different demographic groups. Figure 4a illustrates one such difference, with a Mann–Whitney U test *p*-value of 5.85 × 10^−14^, highlighting how demographic factors can influence kidney function and DFU severity. Elevated ACR levels, indicating potential kidney dysfunction, were found to be associated with higher DFU severity, suggesting a key relationship between renal health and DFU outcomes across these groups. This substantial difference highlights the key molecular and protein compositions that may vary between these groups. Chronic kidney disease (CKD), a common complication of diabetes, signals a progression of DFUs when present. The use of the ACR as a severity factor allows us to explore and quantify the statistically significant differences in kidney function between Hispanic and non-Hispanic populations. Additionally, when evaluating the relationship between vital status (alive vs. deceased) across different demographic groups, Figure 4b shows that deceased patients generally had significantly higher ACR values compared to those who were alive (*p* = 0.0021 and 2.78 × 10^−7^ in Hispanic and non-Hispanic, respectively). This underscores the potential of the ACR as a critical indicator of disease severity and mortality risk, irrespective of demographic background. However, the impact of elevated ACR levels appears to be more pronounced among Hispanic patients (Figure 4).

Several studies support the utility of the ACR in understanding ethnic disparities in kidney-related complications, especially in the context of diabetes. For example, Lawrence et al. [18] demonstrated a significant association between diabetes-related lower-extremity complications, such as amputations, among Hispanic and non-Hispanic populations. In their study, Mexican Americans had a higher incidence of amputations compared to non-Hispanic white patients, with rates of 7.4 per 1000 versus 4.1 per 1000, respectively. Additionally, the amputation-to-ulcer ratio was higher among Mexican Americans (8.7%), suggesting that kidney and lower-extremity complications progress more aggressively in this population. Since kidney dysfunction is often linked to diabetes complications, including DFU severity, the elevated ACR values observed in Hispanic populations indicate a potential factor contributing to these poorer outcomes. Further, Carmen et al. [19] reported that Hispanics exhibit a higher occurrence of albuminuria, a condition characterized by excessive albumin in the urine, compared to white patients. They also found that the Albumin/Creatinine Ratio was significantly elevated in Hispanics relative to other populations. Albuminuria, particularly as measured by the ACR, is a well-established early indicator of kidney damage and a marker of systemic vascular dysfunction, making it a valuable tool for stratification of patients at risk of CKD and DFUs.

These findings highlight the clinical relevance of the ACR as a factor for identifying kidney function and diabetes-related complications. The higher incidence of albuminuria and elevated ACR levels in Hispanics, along with their increased risk of severe complications like amputations, supports the use of the ACR as a sensitive marker. Given the strong statistical differences observed in our analysis, the ACR was shown to be an effective tool for assessing kidney function, making it valuable for early detection, risk stratification, and targeted interventions in populations at risk of severe DFUs.

In this analysis, we further investigated the factors differentiating high and low ACR values by segmenting the ACR data based on the median value of all measurements from the EHRs. This method enabled us to leverage machine learning techniques to assess feature importance, helping us pinpoint the most significant predictors of high ACR levels.

### 2.2. Analysis Using Bulk RNA Dataset

In our analysis of the blood test results from the electronic health records (EHRs), which primarily measure protein concentrations, we aimed to correlate our findings with publicly available RNA-sequencing datasets, a method that provides an averaged gene expression profile across entire tissue samples. The goal was to identify molecular factors for severe DFUs. Our dataset included three stages of disease progression: control (healthy tissue), healing DFU tissue (from DFU patients showing healing progression over a 12-week period), and non-healing DFU tissue. By examining publicly accessible bulk RNA datasets, we observed a significant decrease in the mean gene expression levels in healthy samples compared to diseased samples. This comparative approach helped identify key factors that differentiate these progression levels. Our methodology aligns closely with the approach outlined by Ran Chen et al. [20], ensuring rigor and consistency in validating the ACR test findings within the EHRs. Analyzing these different tissue categories gave us critical insights into the genes and protein markers influencing DFU progression.

A major finding from this analysis was the identification of the *APOE* gene as a significant distinguishing factor. Our results showed that *APOE* expression decreased markedly from the control (117.72) to healing (27.57) and further to non-healing samples (15.83). The Kruskal–Wallis test statistic of 13.18 and a highly significant *p*-value of 0.0014 suggest that *APOE* plays an essential role in the progression of DFUs, particularly in relation to non-healing ulcers. Previous studies, such as those by Xuan He et al. [21], have shown that the *APOE* gene is linked to the ACR, indicating a genetic connection between lipid metabolism and kidney function. Individuals with certain *APOE* alleles are more prone to both dyslipidemia (abnormal lipid metabolism) and kidney damage, underscoring the importance of considering genetic factors in clinical assessments [22,23].

The higher *APOE* expression in healthy compared to non-healing tissue indicates a possible disruption in lipid metabolism and immune regulation in more severe DFU cases. This disruption could be influenced by various demographic factors, such as age, sex, or socio-economic status, which may exacerbate the impact of genetic predispositions like *APOE* allele variations on disease progression. Given *APOE*’s role in lipid metabolism and its connection to cardiovascular health [24], its decreased expression in non-healing DFUs may reflect disrupted lipid metabolism, contributing to poor wound healing and kidney complications in diabetic patients.

While there were significant differences in *GATM* and *CKMT2* expression (0.0077313 and 0.0034734, respectively), there was no clear trend between the control, healing, and non-healing samples. For instance, *GATM* expression sharply dropped from control (55.80) to healing (8.33) and remained relatively low in non-healing (9.67), while *CKMT2* decreased from the control (4.13) to healing (0.82) and remained similar in non-healing samples (0.73). This lack of a clear, progressive pattern from control to non-healing samples limits these proteins as markers of wound healing progression. In contrast, *LDLR* expression showed a more consistent trend across the groups, increasing from the control (70.72) to healing samples (92.86) and reaching the highest level in non-healing samples (118.60). Although the *p*-value for *LDLR* expression (0.1552) does not indicate statistical significance, this gradual progression suggests a potential role in disease severity that may warrant further investigation. Notably, *APOE* is the only gene that showed both statistical significance (*p* = 0.0014) and a clear, progressive decrease in expression. The data for these genes, along with data for other genes, can be found in Table 2.

The observation of significant decreases in gene expression, particularly for APOE, as the tissues transitioned from healing to non-healing states led us to hypothesize that distinct microenvironmental changes are occurring in non-healing DFU cases. To test this hypothesis, we employed single-cell RNA sequencing to identify specific cell types expressing *APOE* and other key genes. The sharp decline in *APOE* expression in non-healing tissues suggests that its role in lipid transport and immune modulation is compromised in more severe DFU cases, contributing to impaired wound healing. Moreover, *APOE*’s involvement in inflammatory responses indicates that reduced expression may reflect an inability to regulate immune processes effectively, potentially leading to poor clinical outcomes in these patients.

### 2.3. Analysis Using Single-Cell RNA Sequencing Dataset

The significant findings from the bulk RNA-seq analysis, particularly the marked decrease in *APOE* expression from healthy to non-healing DFU tissues, prompted us to further investigate the cellular context of this gene’s expression using single-cell RNA-sequencing (scRNA-seq) data. While bulk RNA-seq provided an overview of the gene expression changes across entire tissue samples, scRNA-seq enabled us to delve into gene expression at the individual cell level, uncovering the heterogeneity within the tissue. To achieve this, we utilized the foot dataset from Theocharidis et al. [25], which comprised samples with four levels of DFU disease progression: control (healthy tissue), diabetic, healing, and non-healing samples.

**Figure 5:**
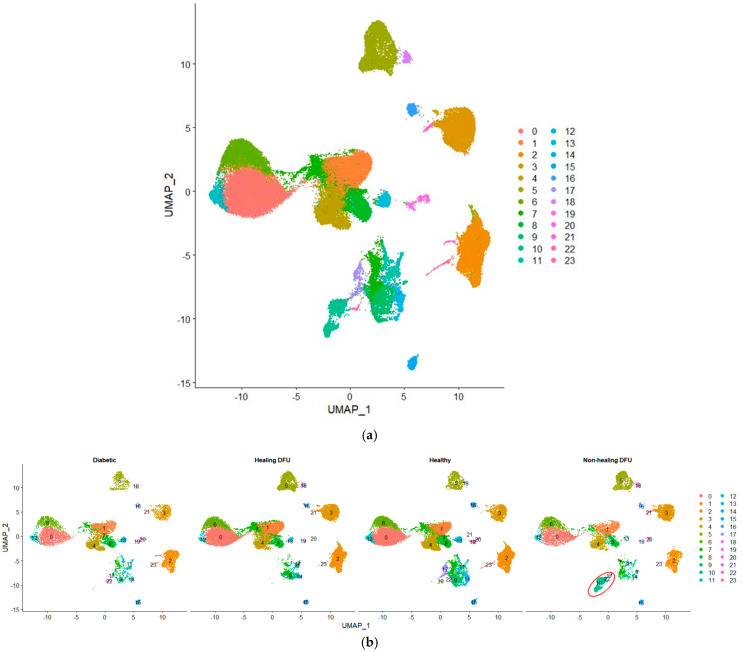
Uniform Manifold Approximation and Projection (UMAP) plot of single-cell RNA sequencing dataset. (**a**) UMAP plot of the integrated dataset, and (**b**) UMAP plot split by sample type.

In our single-cell analysis, we first performed quality control and data normalization to ensure that the gene expression profiles were accurate and comparable across different cell populations. We then applied clustering using the Seurat package, which groups cells with similar gene expression patterns, allowing us to identify and label distinct cell populations. Figure 5a shows the Uniform Manifold Approximation and Projection (UMAP) plot visualizing 24 clusters from our single-cell RNA-sequencing (scRNA-seq) data. Figure 5b compares the cell cluster distributions across four tissue conditions: diabetic, healing DFU, healthy, and non-healing DFU tissues. The UMAP projection highlights the differences between these conditions, with some clusters (e.g., clusters 0, 3, and 5) appearing in all tissue types while others are specific to certain conditions. For example, cluster 2 was present mainly in diabetic and non-healing DFU tissues.

A key finding is cluster 10, which is entirely unique to the non-healing DFU sample. As shown in the plot (circled in red), cluster 10 was concentrated specifically in non-healing DFU tissues, suggesting that this group of cells plays a crucial role in non-healing environments. The absence of this cluster in the other conditions (diabetic, healing DFU, and healthy tissues) suggests that these cells may be involved in pathological processes unique to chronic, non-healing ulcers, such as impaired tissue repair or persistent inflammation.

Cluster 10 could represent a specific cell type or state that contributes to impaired wound healing, such as dysfunctional immune cells, fibroblasts, or keratinocytes failing to promote effective tissue repair. The distinct gene expression profile of this cluster likely reflects key molecular pathways involved in persistent inflammation or fibrosis, which are hallmarks of non-healing DFUs. Understanding the specific role of these cells is critical, as they may be driving the chronic nature of the ulcers by disrupting normal wound-healing processes. To further explore the characteristics of cluster 10 and identify its likely cell type, we employed the SingleR method, a robust cell annotation tool that assigns cell identities by comparing the gene expression profiles of our clusters to reference datasets of known cell types.

**Figure 6:**
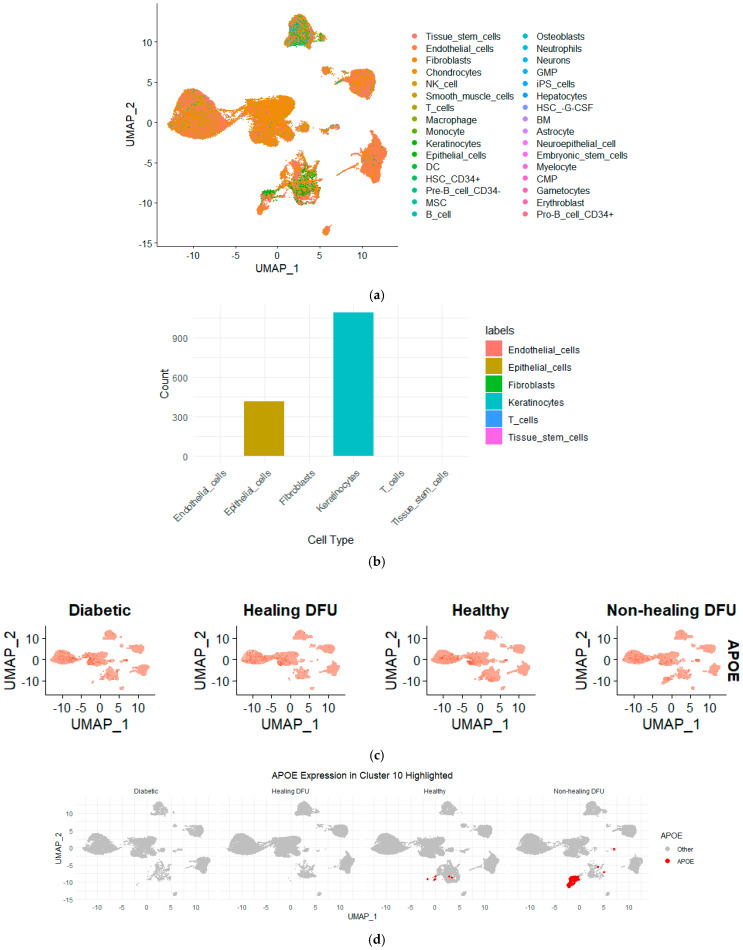
(**a**) UMAP projection showing the distribution of various cell types across the non-healing DFU sample. Different colors represent distinct cell types, demonstrating the distribution and clustering of cell populations across the dataset. (**b**) Bar plot representing the cell type composition in cluster 10, with keratinocytes forming the majority, followed by epithelial cells. (**c**) UMAP plots showing the expression of *APOE* across the different tissues. Higher *APOE* expression was observed in non-healing DFU samples, particularly in specific clusters, as indicated by the intensity of the red color. (**d**) UMAP highlighting *APOE* expression, specifically in Cluster 10. Increased *APOE* expression was prominently visible in non-healing DFU tissues, suggesting its potential role in disease pathology.

Figure 6 illustrates that keratinocytes made up the majority of the cells in cluster 10, with additional cell types, including epithelial cells and fibroblasts, also present. This suggests that keratinocytes, which are vital for maintaining the skin barrier and promoting wound healing, are likely dysfunctional in non-healing DFU environments since they form a distinct cluster from rest of keratinocytes. The presence of epithelial cells and fibroblasts, which are also key players in tissue repair and regeneration, further highlights the complexity of impaired healing in non-healing DFUs. The dysfunction of these cell types could be driving the chronic inflammation and fibrosis associated with non-healing ulcers. These findings strongly support the hypothesis that compromised keratinocyte function, alongside disrupted epithelial and fibroblast activity, may be contributing to the persistence of the non-healing state in DFUs.

To further validate our bulk RNA-seq analysis findings, we queried the single-cell RNA-seq dataset for the genes identified as differentially expressed in the bulk RNA sequencing analysis, focusing on *APOE*. By mapping the expression of *APOE* across the clusters in the single-cell dataset, we found that *APOE* was expressed in cluster 10. This localization of *APOE* to keratinocytes in the non-healing DFU tissue underscores the potential role of *APOE* in the impaired function of these cells. Keratinocytes are essential for skin integrity and wound healing, and the downregulation of *APOE* in this cell type may contribute to the chronic inflammation and poor healing observed in non-healing DFUs. The combined use of bulk and single-cell RNA-seq allowed us to confirm the gene expression changes at the tissue level and pinpoint the specific cell types, such as keratinocytes, where these changes are most relevant in driving the disease pathology.

Finally, we conducted a differential gene expression (DGE) analysis between cluster 10 and the other clusters to identify the genes uniquely enriched in this cluster. This analysis provides a clearer picture of the specific genes present in cluster 10 and highlights distinct biological pathways that may explain its unique characteristics compared to other clusters in the sample. Understanding these pathways is crucial for uncovering the molecular mechanisms contributing to the dysfunctional state of keratinocytes and other cells within cluster 10, likely driving the impaired wound healing observed in non-healing DFUs. By identifying these key pathways, we gain deeper insights into the factors differentiating cluster 10 from other cell populations in the tissue and how these differences may contribute to the chronic, non-healing state of DFUs.

Differential gene expression analysis between cluster 10 and all other clusters identified 231 genes differentially express in cluster 10 (adjusted *p*-value < 0.05). Notably, we observed genes such as *APOE, KRT14*, and *COL1A1* suggesting dysregulated activity in lipid metabolism, keratinocyte function, and extracellular matrix organization. Functional enrichment analysis revealed that these genes are involved in pathways related to wound healing, inflammatory response, and skin development.

## 3. Discussion

The findings of this study provide several key insights into the molecular mechanisms underlying diabetic foot ulcers (DFUs). Starting with the use of EHRs, we identified the Albumin/Creatinine Ratio test as a key indicator, particularly in the context of albumin and creatinine as indicators of kidney function and disease progression. The Albumin Creatinine Ratio (ACR), which we utilized as a risk indicator, is a well-established measure for assessing kidney function and detecting early kidney damage, a critical complication in diabetes [26,27]. Albumin, produced by the liver, plays a vital role in maintaining the oncotic pressure within blood vessels, preventing fluid leakage into surrounding tissues. Consequently, deviations in albumin levels are often indicative of liver or kidney disorders, both of which are common complications associated with DFUs.

Recent studies have highlighted the importance of albumin levels in predicting DFU risk, with significant correlations between reduced albumin levels and heightened risks of severe complications, including non-healing ulcers [28]. Meanwhile, ACR serves as a marker for kidney function as it is a waste product filtered by the kidneys. Elevated creatinine levels typically suggest impaired kidney function, reinforcing the relevance of using the ACR as a robust measure of DFU risk [29]. Our analysis of the ACR as a risk index across different demographic groups provides insights into how biological factors may influence chronic disease progression and DFU severity. Understanding these demographic variations is crucial for developing more equitable and effective healthcare strategies. The Mean Decrease in Accuracy (MDA) metric further validated the ACR, with the associated blood tests grouped into key categories such as lipid metabolism, white blood cells, and red blood cells.

Lawrence et al. [18] have already performed extensive work to show a significant link between Mexican American and non-Hispanic white patients regarding this disease. We examined the Albumin Creatinine Ratio as an indicator to distinguish between the Hispanic and non-Hispanic populations. The ACR, a crucial measure in assessing kidney function and potential kidney damage, provides insights into the overall health factors associated with these populations. This analysis is crucial for understanding the broader implications of ethnic differences in chronic disease, particularly for conditions like diabetes that heavily impact kidney health.

The other blood tests assessed using the MDA also validated the ACR. They can be generally grouped as tests for lipids (Low-density lipoprotein- (LDL) and very Low-density lipoprotein (VLDL) cholesterol), metabolism (calcium), white blood cells (neutrophils/100 leukocytes), and red blood cells (erythrocyte count). Recent studies have shown a significant link between the ACR and dyslipidemia, which is characterized by abnormal lipid levels [21,30,31]. High ACR levels are frequently associated with elevated triglyceride and LDL cholesterol levels, and reduced HDL cholesterol levels. These lipid abnormalities contribute to the progression of cardiovascular diseases, underscoring the interconnectedness of kidney and cardiovascular health.

Lipid tests (LDL and VLDL cholesterol) were particularly important, as they highlighted a significant association between the ACR and dyslipidemia, which is characterized by elevated triglycerides and abnormal cholesterol levels. These findings suggest that lipid metabolism and kidney function are closely linked, and that demographic factors such as age, diet, and lifestyle may affect this relationship, influencing the risk and severity of DFUs. This observation aligns with studies demonstrating a connection between abnormal lipid levels and both cardiovascular disease and kidney dysfunction, underscoring the complex interplay between kidney health and cardiovascular risk as it pertains to complications of DFUs [32]. The enrichment of lipid metabolism genes, including *APOE,* further emphasizes the role of lipid regulation in DFU progression, with recent findings linking *APOE* alleles to abnormal lipid metabolism and kidney damage. The bulk RNA analysis revealed that *APOE* expression was significantly reduced in non-healing DFU tissues compared to healthy and healing DFU tissues. This downregulation of *APOE* suggests a disruption in lipid metabolism, which may impair wound healing and exacerbate chronic inflammation in non-healing DFUs.

The pathway analysis using the Kyoto Encyclopedia of Genes and Genomes (KEGG) database identified several key biological pathways that provide additional insight into the molecular processes associated with DFUs. Pathways related to immune responses and infections were significantly enriched, including those involved in Salmonella infection, legionellosis, and pathogenic Escherichia coli infection. The high representation of these pathways suggests that immune responses and bacterial infections are particularly active in the sample, which aligns with the known immune dysregulation observed in chronic wounds like DFUs. Chronic inflammation, driven by both immune cell dysfunction and persistent bacterial infections, is a hallmark of non-healing wounds [33,34]. These findings reinforce the idea that unresolved infections play a pivotal role in impairing wound healing, likely contributing to the non-healing state observed in some DFUs [35].

In addition to infection-related pathways, significant enrichment was observed in cell signaling pathways such as the gap junction and estrogen signaling pathways. These pathways are integral to intercellular communication and tissue homeostasis, which are disrupted in chronic wounds. The enrichment of cancer-related pathways such as small cell lung cancer pathways and pathways in cancer indicates that cellular proliferation, survival, and apoptosis pathways may be dysregulated in non-healing DFU tissues. This dysregulation suggests that processes normally involved in tissue regeneration may be impaired, contributing to the chronic, non-healing state of DFUs [36].

One of the most significant findings from the single-cell RNA sequencing (scRNA-seq) analysis was the identification of cluster 10, a distinct group of cells present almost exclusively in non-healing DFU tissues. Further analysis revealed that keratinocytes formed the majority of this cluster, alongside smaller populations of epithelial cells and fibroblasts, as shown in Figure 6. These cell types are critical for maintaining the skin barrier and promoting tissue repair, suggesting that their dysfunction in cluster 10 could be contributing to the impaired wound healing observed in non-healing DFUs.

The differential gene expression analysis between cluster 10 and other clusters provided additional insights into this population’s unique gene expression profile. The downregulation of genes involved in cell migration, lipid metabolism, and immune responses suggests that these keratinocytes are not only dysfunctional but may also contribute to a pro-inflammatory environment that hinders healing. The use of the SingleR tool for cell type annotation allowed us to further confirm the identity of cells within cluster 10, providing a clearer understanding of how these cells contribute to non-healing DFU environments.

While this study provides valuable insights, it is essential to acknowledge certain limitations. Firstly, the electronic health record (EHR) dataset contained incomplete or missing data for specific laboratory tests, potentially compromising the robustness of our analysis and introducing inherent bias into the results. Furthermore, the lack of standardization in test ordering practices among patients limited the generalizability of certain conclusions. Secondly, the retrospective nature of the analysis restricts our capacity to definitively establish causality between the identified biomarkers and disease progression.

In conclusion, our study sheds light on the complex molecular mechanisms underlying DFU progression and identified key factors involved in the development and worsening of the disease. By utilizing a multimodal approach that incorporated EHRs, and bulk RNA-seq and single-cell RNA-seq datasets, we highlighted the crucial roles of inflammation, immune responses, lipid metabolism, and eventual cell dysfunction in DFUs. The integration of these datasets allowed us to pinpoint key molecular markers, such as *APOE*, and to gain deeper insights into the specific cell populations driving the chronic, non-healing state of DFUs. These findings provide valuable therapeutic targets for improving wound healing outcomes, particularly for patients at high risk of developing chronic, non-healing ulcers. Future research will further explore the roles of *APOE* and other key genes in DFU progression, and leverage advanced transcriptomics techniques, such as spatial transcriptomics, to investigate potential interventions aimed at modulating these pathways and promoting tissue repair.

## 4. Materials and Methods

### 4.1. Data Source

The dataset utilized in this study was sourced from the OCHIN database [37,38]. The OCHIN database has been utilized and referenced by over 300 research works, dating back to its earliest known application in 2007. Approximately 26 of these studies are specifically focused on diabetes-related research. The contributions of other research teams, such as Chamine et al. [39] and Gemelas et al. [40] who also leveraged the OCHIN database, has significantly enhanced our knowledge of healthcare outcomes and patient populations, shedding light on various medical conditions and how quality datasets can advance the field of healthcare research. After data acquisition, we filtered the data to ensure data integrity in the rest of the studies. This study was conducted in compliance with the ethical principles outlined in the Declaration of Helsinki. The Declaration sets forth guidelines for medical research involving human subjects, emphasizing respect for individuals, the need to obtain informed consent, and the protection of patient rights. Ethical approval for this study was obtained from UTSA (IRB Number FY22-23-75). All data extracted from electronic health records (EHRs) were anonymized to ensure patient confidentiality and compliance with institutional and national ethical standards.

**Figure 7:**
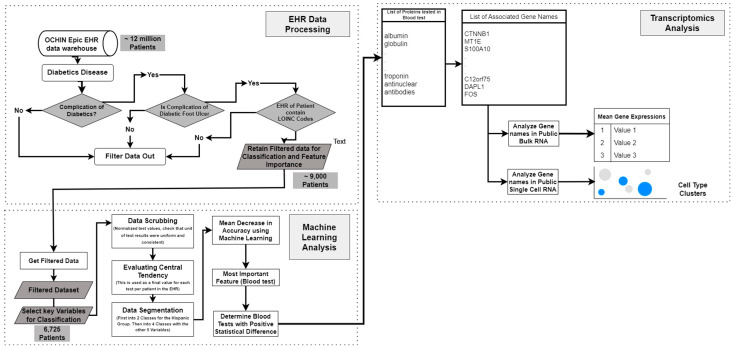
Workflow of integrating EHR data and transcriptomics analysis for diabetic foot ulcer study.

The steps taken are detailed in Figure 7 below.

### 4.2. Data Preprocessing

Because the dataset was pooled from several medical facilities, there is a likelihood of high variance in LOINC code entries due to varying units of measurements or data entry protocols [41]. Careful and thorough data scrubbing was carried out to ensure that this was not an issue. We evaluated the normal range for each test, cross-referencing with recorded lab tests. We evaluated the median value for each of the LOINC codes for each test and used this value to determine a threshold beyond which, the test results were considered an error. Test results with human errors were excluded. Overall, about 5% of the test results were excluded.

After this process, we carried out data segmentation to apply machine learning algorithms to the dataset. We especially checked for data imbalances after segmentation and significant differences to ensure the validity of our findings. We used the Random Forest machine learning algorithm to determine the Mean Decrease in Accuracy.

### 4.3. Machine Learning

Random Forest is a popular machine learning algorithm known for its versatility and robustness. It is based on the concept of ensemble learning, where multiple decision trees are combined to make robust predictions. Random Forest offers exceptional performance in various tasks such as classification, regression, and anomaly detection. The algorithm operates by constructing a multitude of decision trees during the training phase, with each tree built using a random subset of the original features and a random subset of the training data, a technique known as bootstrap aggregation or “bagging” [42]. These individual trees collectively form a “forest”, where the final prediction is made by averaging the results of all the trees (for regression) or by majority vote (for classification).

In this study, the Random Forest algorithm was implemented using the Scikit-learn library in Python. The model was trained using default hyperparameters, including 100 trees (n_estimators = 100) and the Gini impurity criterion for node splitting. The data were split into training and test sets, with 80% used for training and 20% for testing. All computations were performed on a computer with an Intel Core i7 processor (3.8 GHz) and 32 GB of RAM, and running Python 3.9. After evaluating the Mean Decrease in Accuracy, laboratory test labels were cross-referenced in the LOINC code database to ascertain protein names and their associated genes, which were then linked to the RNA datasets for further analysis. [43]

### 4.4. Transcriptomics

In this study, we utilized the bulk RNA-seq dataset previously published by Ran et al. [20], which consisted of eight healthy samples, seven diabetic healing DFU samples, and six diabetic non-healing samples. The processing steps were as prescribed in the paper. The data was normalized using transcripts per million (TPM).

Additionally, we incorporated a single-cell RNA-seq dataset, which enables high-resolution profiling of gene expression at the individual cell level. The dataset used in this study was previously published by Georgios Theocharidis et al. [44]. It consists of eight healthy patients, six diabetic non-DFU patients, seven diabetic healing DFU patients, and four diabetic non-healing DFU patients. To process the single-cell RNA-seq data, we employed a set of computational methods and deep learning tools. First, quality control was performed to filter out low-quality cells based on the number of detected genes, percentage of mitochondrial expression, and unique molecular identifiers (UMIs). Next, normalization and scaling were conducted to account for the sequencing depth and technical variability between cells. In this step, we used the SCTransform function provided by the Seurat R package, which is based on regularized negative binomial regression.

As a third step, dimensionality reduction techniques including Principal Component Analysis (PCA) and Uniform Manifold Approximation and Projection (UMAP) were applied to reduce the dimensionality of the data by preserving the variance and global structure. This was followed by clustering using the Leiden algorithm implementation in Seurat, a graph-based clustering algorithm that was used to identify groups of cells with similar gene expression patterns.

Finally, the sample data were integrated in two steps. First the data from samples from the same severity type were integrated together. Then, the data from the samples with the four different degrees of severities were integrated. This was performed through the Canonical Correlation Analysis (CCA) method provided by the Seurat R package. After the data integration, we used the SingleR Cell-Type Annotation package to annotate the clusters and perform further downstream analysis.

## Figures and Tables

**Table 1:** A table of laboratory tests that show statistically significant differences between Hispanic and non-Hispanic groups. The *p*-value was evaluated using the Mann–Whitney U test.

Feature	Normal Values	Mean (Hispanic)	Mean (Non-Hispanic)	Median (Hispanic)	Median (Non-Hispanic)	*p*-Value (Mann–Whitney U Test)
Alkaline phosphatase	44–147 IU/L	107.74	99.64	96.5	90.5	1.26 × 10^−14^
Albumin/creatinine (U) [Mass ratio] in urine	0–30 mg/g	746.78	454.4	130	64.375	5.85 × 10^−14^
Urea nitrogen/creatinine	10–20	20.76	18.88	20	18	2.7 × 10^−18^
Coagulation tissue factor-induced	0.8–1.1	2	2.99	1.2	2.65	0.000986
Albumin/globulin [ratio] in blood	1–2	1.34	1.38	1.35	1.375	0.00022
Monocyte count	0.02–0.08	0.56	0.62	0.52	0.5845	2 × 10^−19^
Glomerular filtration rate	90–120	86.71	81.18	91	82.5	9.9 × 10^−13^
Erythrocyte mean corpuscular hemoglobin concentration	32–36	32.99	32.79	33.15	32.9	2.57 × 10^−9^
Erythrocyte count	4.2–6.1	4.364	4.48	4.375	4.5	1.16 × 10^−11^
Platelet mean volume	8–12	10.48	10.32	10.4	10.3	0.000266
Lymphocytes/100 leukocytes	20–40%	26.80	26.37	26.6	25.875	0.00306
Neutrophils	2500–7000	3294.91	2339.42	3893.5	52	0.000549

**Table 2:** Transcripts per million (TPM) counts in bulk RNA dataset for a gene associated with a significant blood test in the diagnosis of DFUs. It also reflects the various degrees of severity of DFUs. *APOE* shows a significant difference in mean expression as the severity increases.

Laboratory Test Parameter	Associated Gene Names	Control	Healing	Non-Healing	Kruskal–Wallis Test Statistic	*p*-Value from Mann–Whitney U Test
Albumin	*ALB*	0.205592661	0.008977797	0.029324667	8.92857	0.0115
Creatinine	*CKM*	0.499565985	0.173955302	0.207658008	3.56275	0.168406596
	*SLC22A12*	0.006241889	0	0.077868848	3.1312025	0.208962
	*SLC22A2*	0.010733984	0.004172606	0.006355688		
	*GATM*	55.79607655	8.327619645	9.674862405	9.7249536	0.0077313
	*CKB*	93.50450349	52.65157717	54.05675365	3.030303	0.2197748
	*CKMT1A*	46.5820514	50.68992477	43.91039501	0.8126159	0.6661049
	*CKMT1B*	36.42784045	37.23741686	36.16401885	0.3178726	0.853050
	*CKMT2*	4.13219376	0.822784598	0.728522893	11.32522	0.0034734
Cholesterol	*LDLR*	70.72075147	92.85914778	118.6011276	3.725572	0.155239
	*PCSK9*	5.25025018	2.867216518	3.207883812	5.454545	0.0653974
	*APOE*	117.7178759	27.56887668	15.82637224	13.18058	0.0013736

## Data Availability

The original contributions presented in the study are included in the article, further inquiries can be directed to the corresponding author/s.
